# Supplementary figures and images for: Hypothalamic representation of the imminence of predator threat detected by the vomeronasal organ in mice
Source: eLife. 2024 Oct 16;12:RP92982. doi: 10.7554/eLife.92982 (PMC11483128; doi:10.7554/eLife.92982)

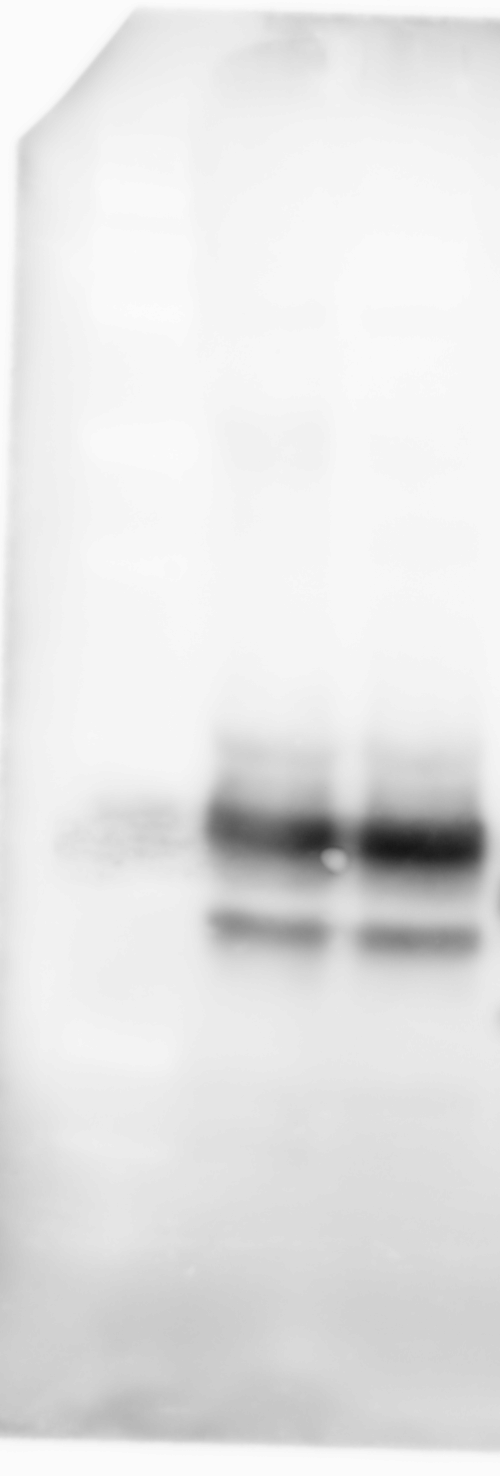

Supplement: Figure 1—figure supplement 2—source data 1. [file elife-92982-fig1-figsupp2-data1.zip › Figure1-S2.tif]

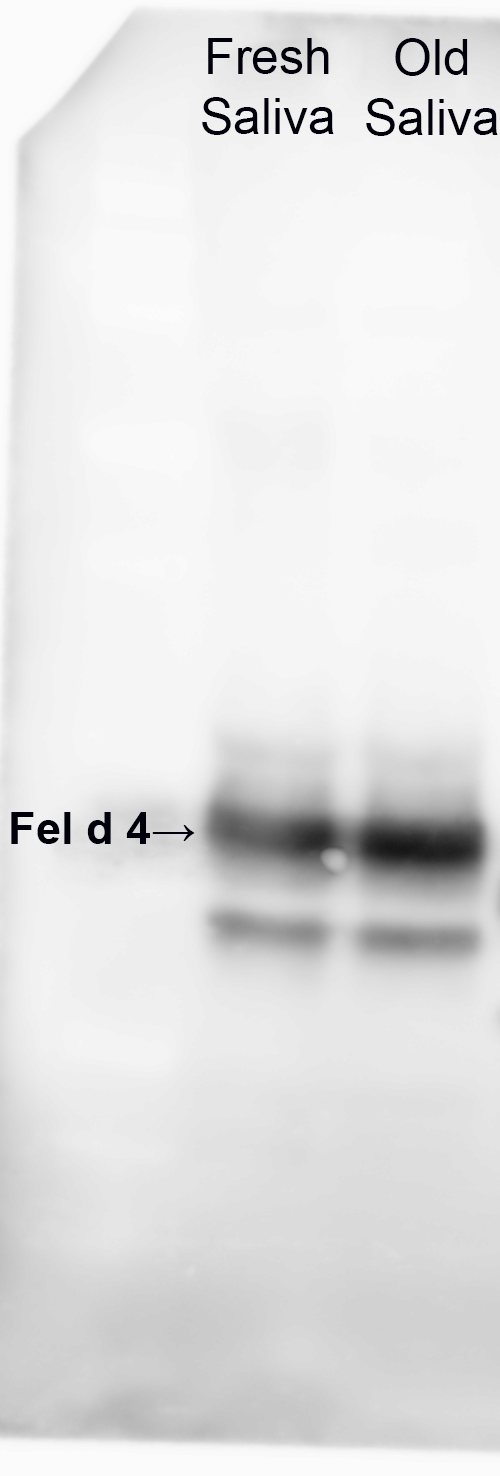

Supplement: Figure 1—figure supplement 2—source data 2. [file elife-92982-fig1-figsupp2-data2.zip › Figure1-S2-labeled.tif]
